# Application of a mechanobiological algorithm to investigate mechanical mediation of heterotopic bone in trans-femoral amputees

**DOI:** 10.1038/s41598-018-32414-1

**Published:** 2018-09-21

**Authors:** Naomi M. Rosenberg, Anthony M. J. Bull

**Affiliations:** 1 Imperial College London, London, SW7 2AZ UK; 2 Present Address: 2 Norrys Close, Barnet, Herts EN4 9JY UK

## Abstract

Heterotopic ossification (HO) is the process of bone formation in tissues that are not usually osseous. It occurs in 60% of those with blast-related amputations. HO can result in reduced range of motion, pain, nerve impingement and can affect prosthesis fitting and is caused by a combination of mechanical, biological, local and systemic factors. As with normal bone formation and remodelling, it is expected that heterotopic bone responds to mechanical stimuli and understanding this relationship can give insight into the pathology. The objective of this research was to investigate whether a physiological 2D computational model that considers both mechanical and biological factors can be used to simulate HO in the residual limb of a trans-femoral amputee. The study found that characteristic morphologies of HO were reproduced by adjusting the loading environment. Significant effects were produced by changing the loading direction on the femur; this is potentially associated with different initial surgical interventions such as muscle myodesis. Also, initial treatment such as negative pressure through a dressing was found to change the shape of heterotopic bone.

## Introduction

Heterotopic ossification (HO) is the process of bone formation in tissues that are not usually osseous. Over 60% of those with severe trauma such as that due to blast develop HO post amputation in the residual limb. The presence of HO in the residual limbs of amputees can result in skin sores and irritation which may lead to the abandonment of a prosthesis by the affected user. Other complications include trapped nerves, infection and reduction or loss of mobility^1,2^. Whilst ectopic bone in the residual limb more often causes complications, in some instances, it can be used to the advantage of the amputee. Melcer *et al*.^3^ noted two cases where heterotopic bone that formed at the distal end of trans-femoral amputees provided an anatomic structure around which the prosthetic socket could be formed thus helping prevent the prosthesis from sliding off. A bony region of tissue has the advantage of stability when being used to fixate a prosthesis unlike soft tissue which changes in volume throughout the day.

Although heterotopic bone often appears chaotic and disorganised^3^, this study aims to investigate whether its global structure may be influenced by the mechanical environment, as has shown to be the case with non-pathological bone^4,5^. Manipulating the structure of heterotopic bone may provide clinical benefit by either reducing the amount of bone produced, by creating a more effective load bearing structure for amputees, or by creating anatomical fixation points to secure prostheses. Loading changes are known to be associated with variable amounts of heterotopic bone production: use of negative pressure dressings has been shown to correlate with increases in HO^6^ and a study by Kir *et al*.^7^ showed that military personnel involved in repetitive firearms exercises (which resulted in multiple impactions from the rifle to the deltopectoral region) were reported to develop heterotopic masses in this region. HO has been seen to develop right up against the skin boundary and in some cases penetrate skin grafts^1,3^. This may indicate that the skin layer has some impact on the progression of HO. A number of studies distinguish a link between tissue trauma and HO and indicate that there is some relationship between the proximity of the development of HO to the site of trauma^8–10^.

Aside from traumatic blast injury, HO is observed in non-traumatic amputation e.g. due to vascular disease (although far less common than in traumatic amputation), various joint arthroplasties and fixations, after spinal cord injury (SCI) and traumatic brain injury (TBI), severe burns, other traumatic injuries not involving blast (such as dislocations or crushing accidents), and in genetic disease^11–20^. In all instances, there is some form of injury and tissue regeneration taking place. Animal studies have shown that subjecting immobilized limbs to sessions of forced manipulation results in HO forming in local regions^21–23^, thus highlighting the combined role of mechanics and trauma.

Previous studies have used a strain energy based method to model the progression of osteophytes^24^ and ectopic bone formation after cervical disc replacement^25,26^. This purely mechanically based model is appropriate as the effects from inflammation and aberrant wound healing may have low influence on this bone formation. This assumption is based on the fact that HO in the cervical region tends to develop over a number of years^27^ and there is less surrounding soft tissue in the region to host inflammation. However, heterotopic bone in amputations has more significant inflammatory effects and is associated with the bioburden induced by the trauma^28^. Therefore, computational modelling of heterotopic bone requires the inclusion of the effects of the biological environment. The aim of this paper is to apply a computational algorithm incorporating mechanobiological factors to the residual limb of a trans-femoral amputee to test the hypothesis that a modification of the mechanical environment can significantly alter both the shape and amount of heterotopic bone produced.

## Materials and Methods

### Geometry, loading, and material properties

**Figure 1:**
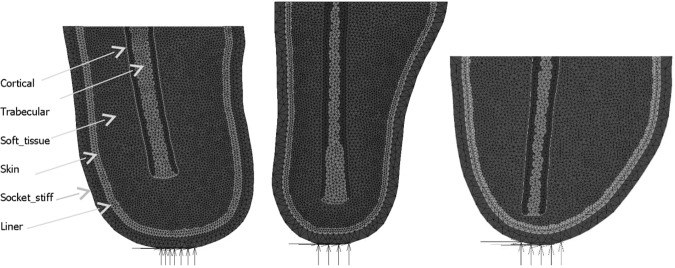
Images of the three different residual stump models.

Three residual stump two dimensional models were created. The outlines of the residual femur were traced from medical images found in the literature and available sources. The geometry of Model 1 was taken from Davis *et al*.^8^, the geometry of Model 2 was taken from Potter *et al*.^29^ and that of Model 3 was taken from an image supplied by Edwards^30^. All models were made of 3 node triangular elements with one central integration point. The models consisted of six materials with a femur consisting of cortical and trabecular bone material that is surrounded by a general soft tissue region. The outer edge of the soft tissue region is made up of a skin layer. The residual limb is then embedded within a liner which is in contact with a socket. The sockets were fixed at the bottom in the *x*, *y* and *z* directions over a span of 3 to 4 cm to represent the distal fixation of the socket to the prosthetic leg and foot (Fig. 1). The finite element analysis was run using Marc Mentat (2015.0.0, MSC Software, US) nonlinear finite element software. Mesh convergence was conducted to produce a compromise outcome of reduced computational time and model detail. The requirement of the model was to reflect the physiological loading environment in a residual limb. Therefore, the minimum detail required was a stiff socket structure, soft tissue and femoral bone. Model parameters are summarised in Table 1.

**Table 1:** Model mesh parameters.

Model	No. of Elements (not including the socket or liner)	Femur width mid-shaft	Proximal width	Area of Soft tissue	Length	Femoral alignment from vertical
Model 1	7864	2.7 cm	13.8 cm	205 cm^2^	20.5 cm	10°
Model 2	6190	2.7 cm	17.4 cm	266 cm^2^	27.7 cm	0°
Model 3	5171	2.5 cm	19.8 cm	233 cm^2^	18.1 cm	−5°

**Figure 2:**
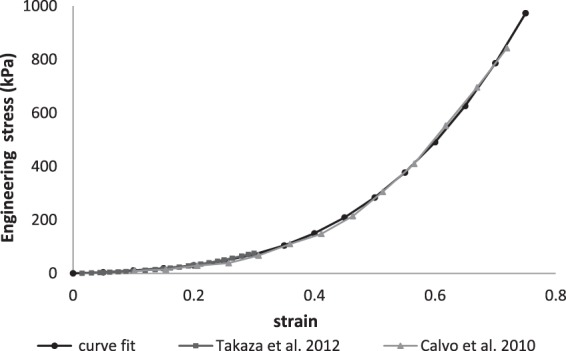
Soft tissue Mechanical Properties used for the FE model.

The liner was given a thickness of 6 mm based on Selinger^31^. The Young’s modulus was set to 1 MPa, taken from the mean value of a range of liners tested in compression^32^. The friction coefficients were taken from tests on polyurethane liners^33^. These were defined as 1.38 between the liner and the socket and as 1.58 between the liner and skin. The Poisson’s ratio was set to 0.49 to act as an incompressible polymer. The socket modulus was set to 15 GPa with a Poisson’s ratio of 0.3^34^ and a thickness of 12 mm^35^. The soft tissue was modelled with a stress strain curve input. Engineering stress strain data was taken from literature in which passive tensile testing on muscle tissue in the longitudinal direction was performed^36,37^ (Fig. 2). The Poisson’s ratio for soft tissue was 0.47^37^. The soft tissue was surrounded by a layer of skin, defined as the outermost layer of soft tissue elements resulting in a thickness of approximately 2–3 mm^38^. No remodelling was permitted to take place within the skin elements.

Boundary conditions were derived from a 2D femoral model^39,40^ to which a remodelling algorithm^41^ was applied with loading applied through the hip and abductor muscles. Loads were applied iteratively until the resulting bone geometry revealed characteristic density distributions (the principle compressive group, principle tensile group and Ward’s triangle) commonly observed in the proximal femur. Then the measured resultant loads through the cortical regions at the proximal femoral level were used as input loads through the residual femur of the trans-femoral stump models. This loading condition is described as upright loading (Table 2) for which the input loads are rotated to align with the long axis of the residual femur for each model. In order to test the effect of different loading conditions and to simulate abducted and adducted gait, the loads were varied by rotating their input angle relative to the femoral long axis by ±30°. Rotating the loads clockwise and anti-clockwise resulted in adduction and abduction of the femur, respectively.

**Table 2:** Baseline loading - “upright loading” - used for the stump models.

Axis	Resultant Medial side (N)	Resultant Lateral side (N)
Horizontal	0	5
Vertical	−237	158

A negative pressure dressing was simulated by applying negative pressure over a span of 10 cm at 26.7 kPa of which is the upper limit provided by most treatment units^42^. The boundary condition was applied in three positions, laterally and medially (midway of the vertical axis) and centrally at the most distal point. The application of a tourniquet was simulated by applying a positive pressure of 40 kPa^43^ over a distance of 3 cm around the centre of the thigh. Therefore, the following simulations were run for all three models: upright, abducted, adducted, negative pressure, tourniquet, reduced skin stiffness and moving the location of trauma from the distal end to the mid lateral region.

### Remodelling

The remodelling algorithm from Mullender *et al*.^41^ was expanded to consider remodelling in soft tissue that was regulated with an extra factor, *β*, that increased with proximity to a specified wound site and with tissue strain^44^. This factor can be used to regulate the density change in each element including soft tissue. If the tissue density is raised passed a certain threshold, the properties are defined to be proportional to the density instead of those defined in Fig. 2. The density threshold was set to be 1.2 g/cm^3^ which is also the maximum density for trabecular bone. The remodelling algorithm causes a change in material density in response to the level of mechanical stimulus and proximity to a specified zone of injury (or maximum level of inflammation). The material properties are then described as a function of the current density (Equation 1).

1 $$\rho {(x)}_{t+{\rm{\Delta }}t}=\rho {(x)}_{t}+\tau \phi {(x)}_{t}\beta {(x)}_{t}$$

In Equation 1, *τ* is a time constant and *φ* is the level of strain energy based stimulus defined in Equation 2 ^41^. *β* is the remodelling parameter introduced in Rosenberg *et al*.^44^ shown in Equation 3.

2 $$\phi (x,t)=\sum _{1}^{N}{e}^{-di(x)/D}(\frac{SED}{\rho }-\,K)\,$$

Equation 2 describes that the stimulus at position *x* at time *t* is the sum of the error between the experienced strain energy and the steady state reference stimulus *K* for every element (from 1 to *N*) where *N* is the total number of elements. The steady state reference stimulus refers to the level of stimulus that results in an equilibrium state of bone turnover. Hence, if the experienced strain energy is equal to the steady state reference stimulus, the change in density will be zero. The exponent in the equation is a spatial influence parameter considering a sensor influence factor *D* and the distance between each element to position *x*. The equation can be expanded to consider a lazy zone^45^.

3 $$\beta ={P}^{(\frac{1}{1.5{\rho }_{MSC}})}$$

In Equation 3, *ρ*_*MSC*_ represents the density of mesenchymal stem cells. This can be considered to be relative to the level of inflammation. In the early stages of injury, there is high proliferation of inflammatory cells and progenitor cells. It may be the elevated levels of inflammation experienced in traumatic blast injuries that enhance the aberrant wound healing process. There are some findings to suggest that inflammatory cytokines promote stem cell activity^46^. The relationship between mesenchymal stem cells in an inflammatory environment and the initiation of HO was considered due to the findings published in the work by Davis *et al*.^8^ who found a significantly higher number of progenitor cells (osteogenic or otherwise) at the wound sites that formed HO. The baseline model assumed that the highest density of mesenchymal stem cells (*ρ*_*MSC*_) is at the level of injury and that this is located at the distal end of the stump models. A linear relationship between the function *ρ*_*MSC*_ and distance to injury point was assumed. The maximum value of *ρ*_*MSC*_ is 1 and the minimum is 0.01. To examine the effect of this function, the peak location was also tested in a lateral location situated slightly proximally to the distal end of the femur. The function of *ρ*_*MSC*_ was then set to decrease linearly in the radial direction. *P* represents the likelihood of ossification based on the current element stiffness and models how substrate stiffness and environment influences cell differentiation. Substrate stiffness affects stem cell lineage and can govern the chance of the progenitor cells differentiating into osteogenic cells^47–49^. The probability function was scaled relative to the Young’s modulus of bone and soft tissue. It was assumed that a cartilaginous environment of approximately 10 MPa^50^ would give a value of *P* just over 10%. Assuming stiffness values from Protopappas *et al*.^51^, a soft callus environment of 1 GPa stiffness was assumed to give a probability factor *P* of 60%, intermediate callus environments of 3 GPa to give *P* = 87% and stiff callus environments to have a probability factor *P* of 100%. If the material was already defined as bone (i.e. the femoral elements in this case), *P* was set to 100%.

Finally, the material stiffness of bone can be found from the current density. The relationship for cortical and trabecular bone was found by collating a range of data from the literature^40^. The relationship for heterotopic bone was set to be the same as that of trabecular bone, however, the range in density for heterotopic bone could reach 2.2 g/cm^3^, exceeding that of trabecular bone (maximum density 1.2 g/cm^3^).

A summary of all the parameters used on the models is shown in Table 3. A more comprehensive study into these different parameters, including a sensitivity analysis, was conducted in previous work by the authors^40,44^. Only the parameters that were found to manipulate the shape of the HO are presented here.

**Table 3:** Parameters used in the baseline stump models.

Parameter	Value	Reference
*K*_*cortical*_	0.0429 *J*/*g*	Derived by assuming levels of steady state stress and strain^44^
*K*_*trabecular*_	0.0125 *J*/*g*	
*K*_*HO*_	0.0029 *J*/*g*	
*K*_*soft tissue*_	0.0029 *J*/*g*	
*ρ*_*cortical initial*_	1.7 *g*/*cm*^3^	^65^
*ρ*_*trabecular initial*_	0.8 *g*/*cm*^3^	^65^
*ρ*_*soft tissue initial*_	0.95 *g*/*cm*^3^	^66, 67^
*ρ*_*cortical range*_	1.2 < *ρ*_*cortical*_ ≤ 2.0 *g*/*cm*^3^	^65^
*ρ*_*trabecular range*_	0.1 < *ρ*_*trabecular*_ ≤ 1.2 *g*/*cm*^3^	^65^
*ρ*_*HO range*_	0.1 < *ρ*_*HO*_ ≤ 2.2 *g*/*cm*^3^	^44^
*ρ*_*MSC*_ range	0.01 ≤ *ρ*_*MSC*_ ≤ 1	
Relation between *E* (GPa) and *ρ*_*cortical*_	*E* = −2.642 + 5.622*ρ* + 0.763*ρ*^2^ + 0.937*ρ*^3^	Relationships derived by collating a range of data and finding the mean trend^44^
Relation between *E* (GPa) and *ρ*_*trabecular*_	*E* = −0.07 + 1.575*ρ* + 0.762*ρ*^2^ + 1.241*ρ*^3^	
Relation between *E* (GPa) and *ρ*_*HO*_	*E* = −0.07 + 1.575*ρ* + 0.762*ρ*^2^ + 1.241*ρ*^3^	
*E*_*soft*,*tissue*_ (*KPa*)	*E* = 0.04 + 124.41*ε* − 479.66*ε*^2^ + 2725.02*ε*^3^	
*E*_*skin*_ (*KPa*)	8000	^68– 70^
*E*_*socket*_ (*MPa*)	15000	^34^
*E*_*liner*_ (*MPa*)	1	^32^
*v*_*bone*_	0.3	^65^
*v*_*soft*, *tissue*_	0.47	^37^
*v*_*skin*_	0.475	^71^
*v*_*socket*_	0.3	^34^
*v*_*liner*_	0.49	Modelled as incompressible
Lazy zone span femur	10%	
Lazy zone span HO	0%	
Sensor influence factor *D*	0.35 *cm*	^41^
Time constant Δt	1	^41^

The steady state stimulus for heterotopic bone was set to be less than that of non-pathological bone to reflect the fact that loading is not directly through the skeletal structure, resulting in lower load transmission. The hypothesis is that although loading magnitude is low, it still influences the progression of HO. HO may be more sensitive to loads as the cells are behaving aberrantly. This is based on the increased rates of bone deposition seen for trauma induced HO^52^ and the higher numbers of osteoblasts and osteoclasts found in ectopic bone^53^. This increased metabolic activity can be represented by decreasing the steady state value which in turn increases the resulting mass of bone in the model. Unlike non-pathological bone which is loaded by surrounding muscle architecture, the environment for HO is chaotic and disorganised, but even small mechanical sensations may influence its otherwise uncontrolled path.

Baseline simulations were run until the changes produced a change in mean absolute density of less than 0.0001 g/cm^3^ per iteration. This occurred by iteration 1250 for the baseline model and was chosen as the end point. Given that traumatic HO is usually ready for excision at 6 months^1,10^, and that normal bone remodelling involves 2 weeks of bone resorption and 4 months to fill the cavity^54^ then the convergence of remodelling may indicate a time point of approximately 5 months.

The negative pressure simulation was run for 115 iterations (corresponding to approximately 2 weeks, representing appropriate clinical use) followed by the standard loading load case for the remainder of the simulation.

The tourniquet loading was applied for one iteration followed by standard loading. The maximum recommended time for tourniquet application is 2 hours^55^. If the full simulation of 1250 iterations is considered to be 5 months, then 1 iteration equates to just under 3 hours.

### Characterisation of Heterotopic Bone

**Figure 3:**
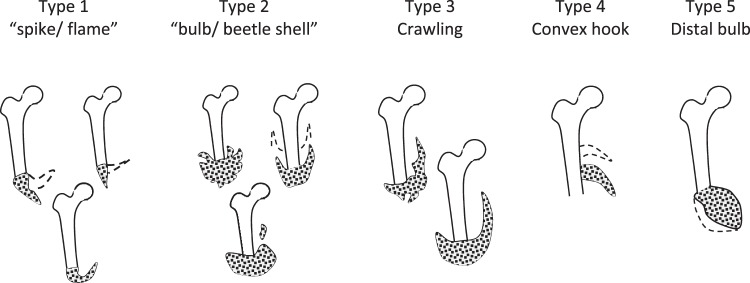
Classifications of heterotopic bone used in this paper.

Medical images were used to define physiological heteropic bone shapes. Evriviades *et al*.^56^ categorised two main types of ossification. Type 1 was described as a flame shaped spike extending from the residual femur and Type 2 as a bulb like beetle’s shell, discontinuous from the residual femur. Another three types were categorised based on radiographs available in the literature^8,29,57,58^. These were classed as Crawling (Type 3), Convex hook (Type 4) and Distal bulb (Type 5). Figure 3 shows visualisations of how the different proposed morphological classifications of traumatic HO may appear in a simplified finite element (FE) model. Dashed lines in the sketches indicate variations in position or volume of the heterotopic bone.

## Results

**Figure 4:**
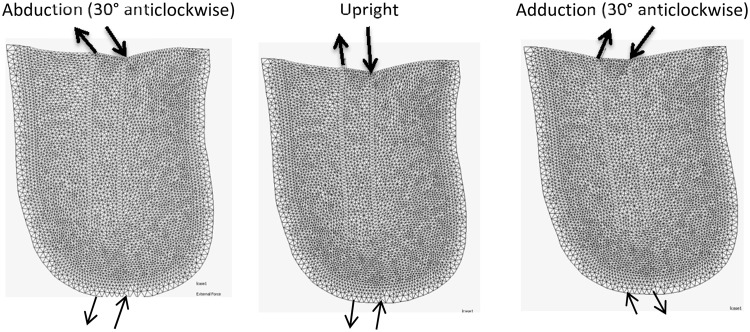
Loading cases applied to the femoral model. The top arrows represent the input load directions and the bottom arrows represent the resultant forces at the fixation nodes.

**Figure 5:**
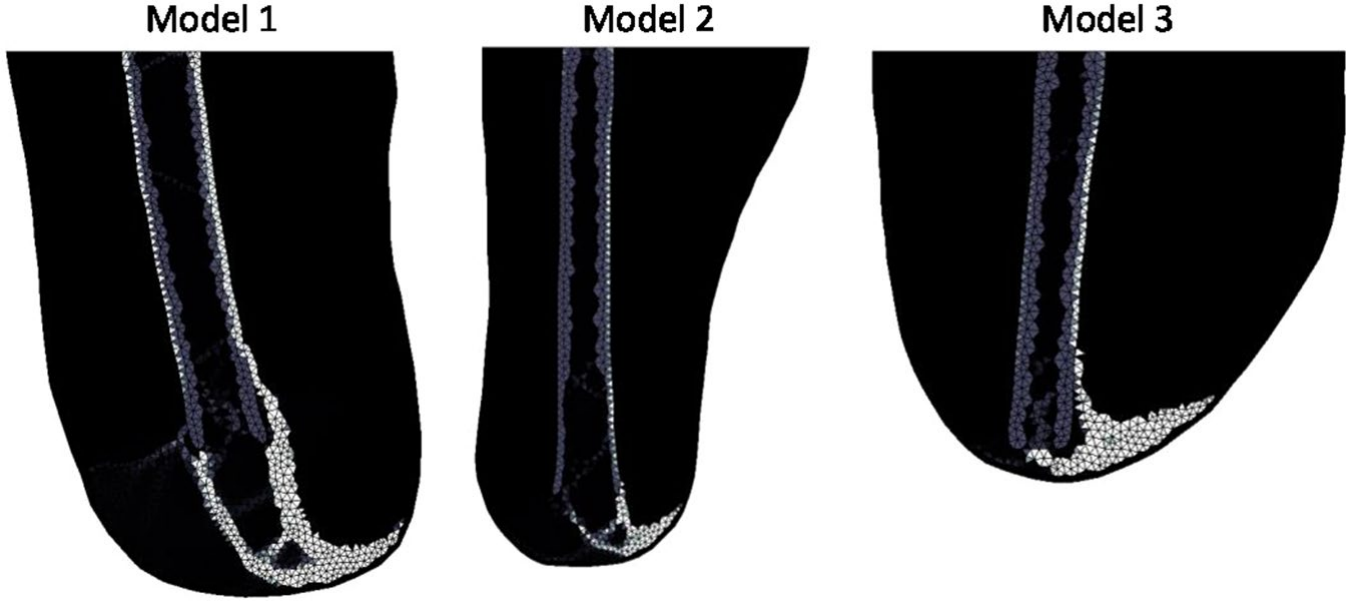
Upright loading stiffness results showing Type 1 morphology. Lighter shading represents higher Young’s Modulus.

**Figure 6:**
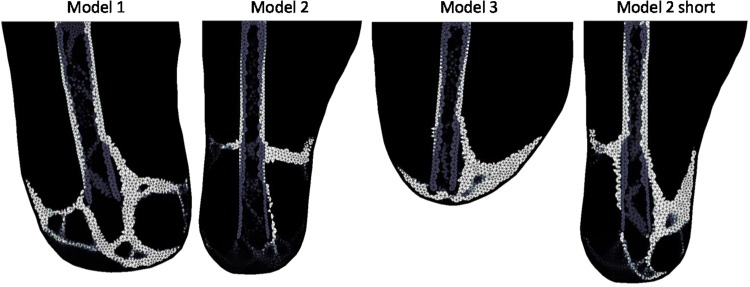
Abducted (30° anticlockwise) loading stiffness results showing Type 2. Lighter shading represents higher Young’s Modulus.

**Figure 7:**
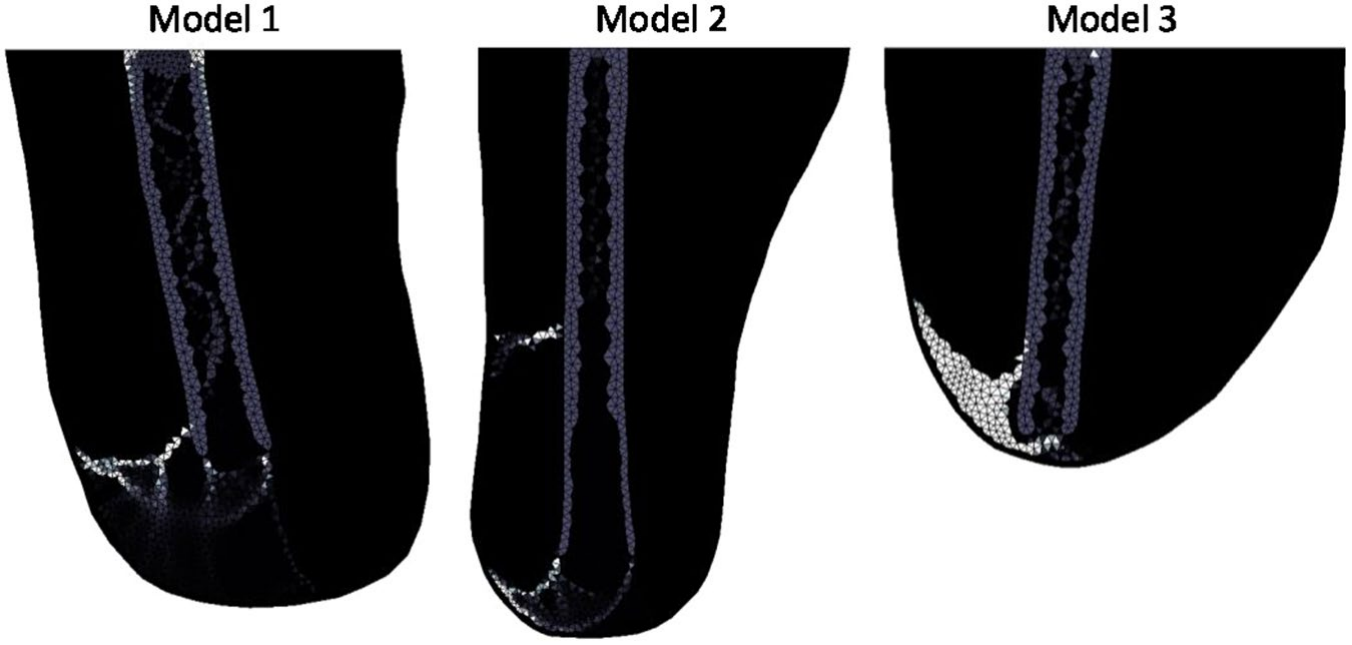
Adducted (30° anticlockwise) loading stiffness results showing non-physiological heterotopic bone morphology. Lighter shading represents higher Young’s Modulus.

The different loading cases are demonstrated in Fig. 4. Upright loading resulted in Type 1 HO (Fig. 5). Abduction tended to result in Type 2 appearing formations of HO (Fig. 6). Model 2 showed a less clear Type 2 pattern, most likely due to the length of residual femur. This was reduced in length by 2 cm resulting in 5 cm of soft tissue distal to the residual femur (the same as for Model 1). The results from this test showed a clearer Type 2 morphology. Adduction loading resulted in lateral facing Type 1 morphologies (Fig. 7). This is not seen in the literature.

**Figure 8:**
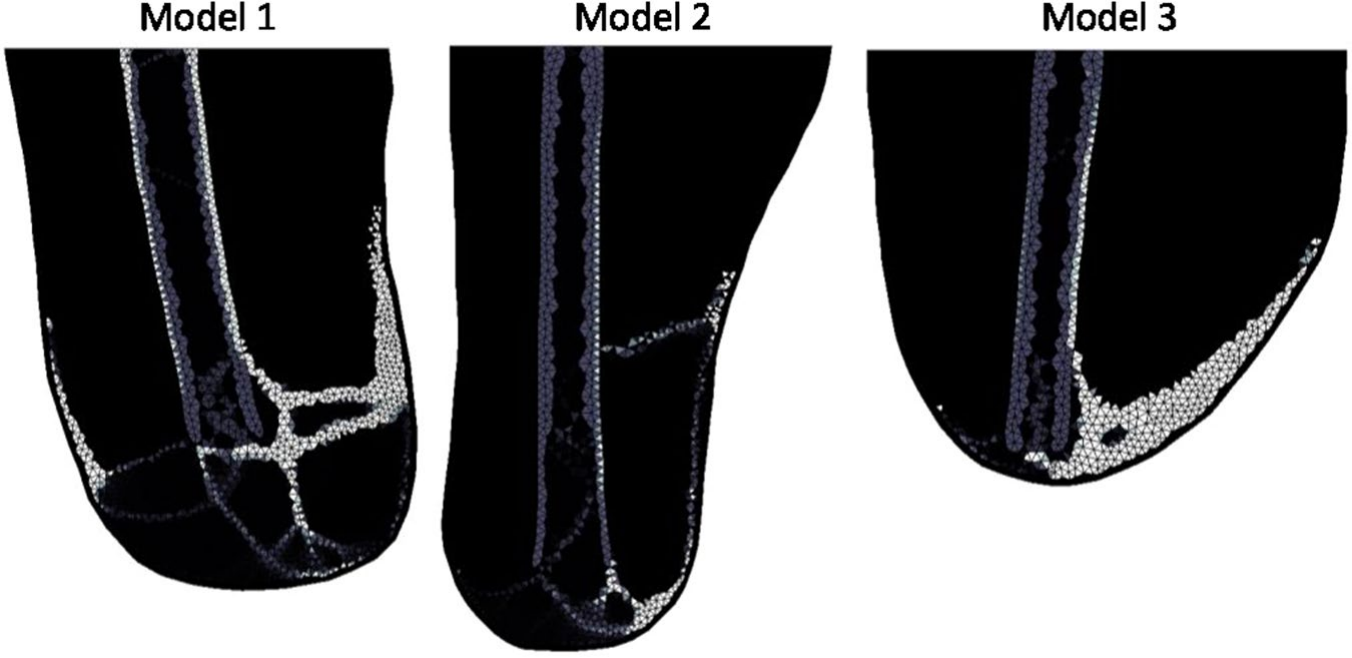
Upright loading stiffness results with skin stiffness reduction to 60kPa showing Type 3. Lighter shading represents higher Young’s Modulus.

Decreasing the stiffness of the skin layer encouraged the Type 3 crawling formation of HO (Fig. 8).

**Figure 9:**
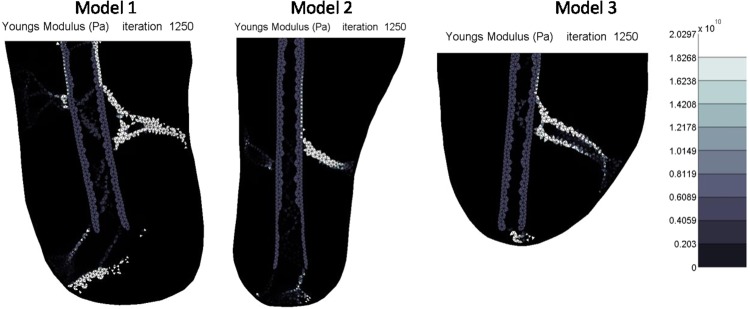
Stiffness results when the peak indicator of inflammation (*ρ*_*MSC*_) was located in the mid lateral region for Models 1 and 2 and a mid medial region for Model 3 showing Type 4 morphology. Lighter shading represents higher Young’s Modulus.

Changing the location of the peak cellular contribution (*ρ*_*MSC*_) resulted in different shapes of ossification. Islands or detached regions of HO tended to emerge in in areas where stimulus was high but *ρ*_*MSC*_ was relatively low, for example near the distal end of the femur in Model 1. The bulk of the HO formed where both stimulus and *ρ*_*MSC*_ was high. This formation tended to resemble the Type 4 convex hook appearance of HO (Fig. 9). The Type 5 distal bulb formation did not occur in these tests. It is assumed that this is due to the available area of soft tissue.

**Figure 10:**
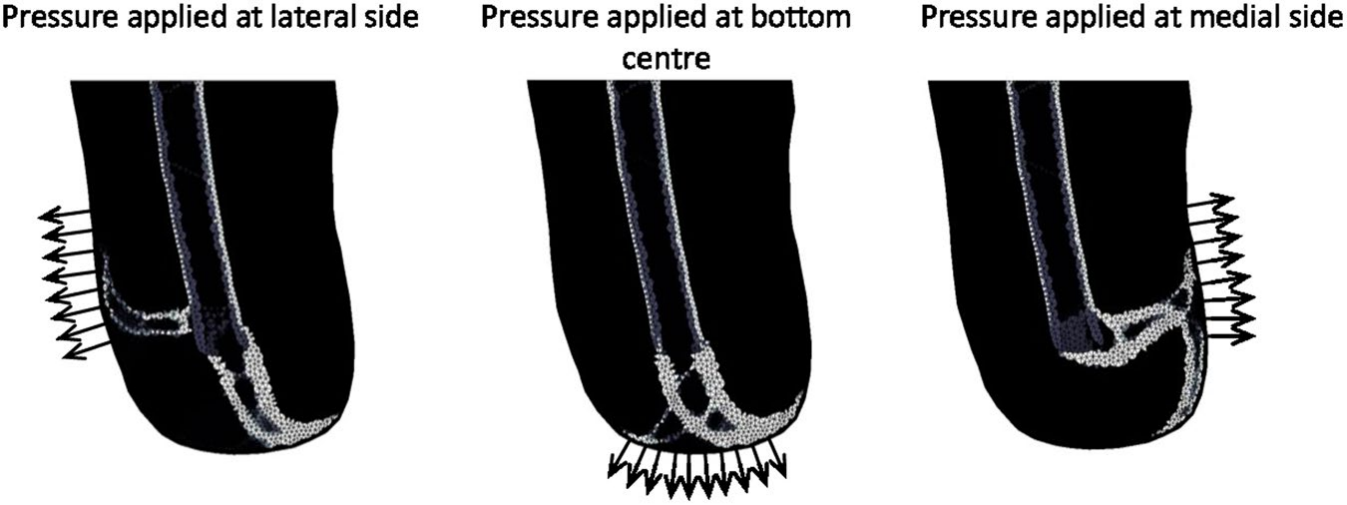
Stiffness results from 1135 iterations of standard loading applied after 115 iterations of negative pressure application. Lighter shading represents higher Young’s Modulus. The direction of pressure applied is indicated by the arrows.

The results of the negative pressure of 26.7kPa, simulating a negative pressure dressing, demonstrated that the pressure dressing could significantly alter the path of HO (Fig. 10).

**Figure 11:**
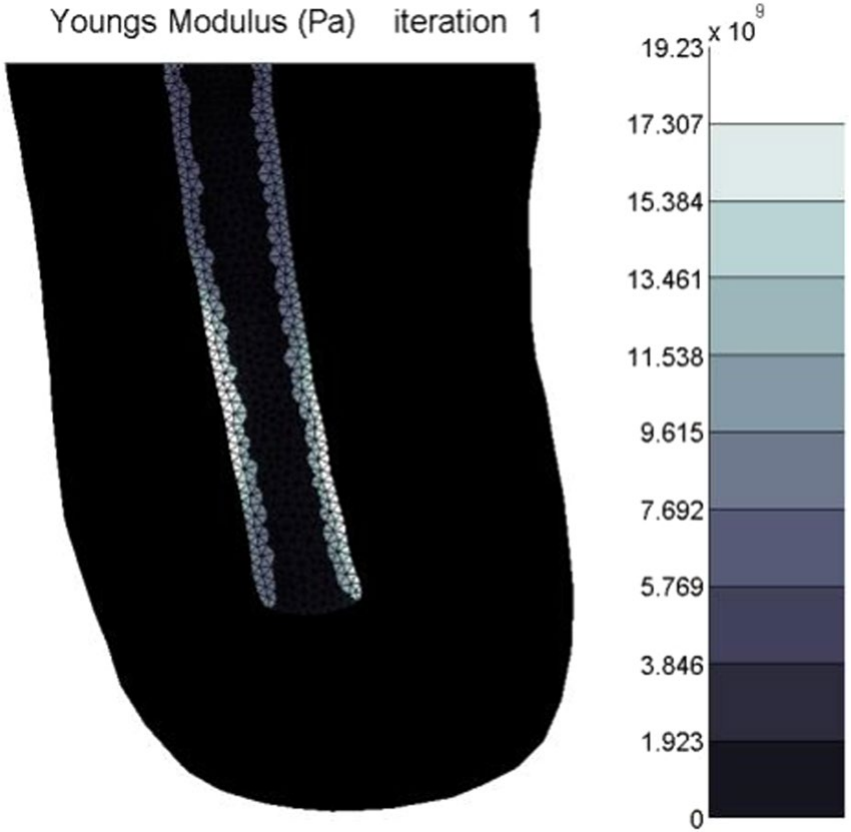
Stiffness distribution after the first iteration with tourniquet application.

Tourniquet loading followed by standard loading resulted in no visible change in the resulting new bone (Fig. 11).

## Discussion

In this study, a computational algorithm incorporating mechanobiological factors for the formation of HO was applied to three 2D trans-femoral stump geometries and demonstrated that modification of the mechanical environment significantly alters both the shape and amount of heterotopic bone produced. Adjusting the direction of load, skin material properties and the location of maximum trauma resulted in four characteristic types of HO. The simulation of negative pressure dressings and a tourniquet application also served to highlight behavioural traits of HO. The results showed that heterotopic ossification is likely sensitive to local loading as different characteristic types emerged under different loading conditions. The mechanobiological algorithm used was based on strain energy and biological parameters: distance to injury location and current element stiffness.

In all instances, the elements which were driven to stiffen were those under the highest absolute strain. The level of strain was predominantly driven by how the femur moved within the soft tissue under the applied load. In the instance of negative pressure simulations, residual stresses in elements in the direction of the applied pressure encouraged these elements to stiffen when loading through the femur was applied.

The Type 1 medial facing hook shapes of HO formed under standard loading. It may be argued that the Type 1 appearance of HO resembles a bone spur or callus that did not join with an adjacent piece of bone. The mechanical environment may be what is responsible for the tendency for the new bone to point medially. Beetle like Type 2 HO was seen at high levels of abduction. Trans-femoral amputees are likely to experience some level of abduction in their gait and this has been noted as a common deviation in gait^59^. The reason for this may be that the muscle bone attachment sites of the hip abductor muscles are more proximal than the adductor muscles and so are less likely to be involved in the injury and surgical transection^60^, whereas the attachment sites for adductors may be damaged or lost in the amputee^61^. The preservation of abductors and loss of adductors naturally results in abduction. Surgeons may perform myodesis to reconnect the remaining adductor muscles to the bone, however, this may not be possible in all cases and the procedure itself may add to the tissue trauma.

Reducing the stiffness of the skin produced HO that crawled up the side of the limb which is also a characteristic trait of HO seen clinically (characterised as Type 3 HO). This suggests that the occurrence of the crawling type of HO may be due to increased strains in the skin that result from the wearing of a prostheses.

Changing the location of maximum *ρ*_*MSC*_ a marker of cellular activity, influenced distribution of HO. In the baseline model where *ρ*_*MSC*_ increased linearly toward the distal end of the model, the stimulus and level of *ρ*_*MSC*_ were high in the same location distal to the residual femur. When the peak location of *ρ*_*MSC*_ is moved, the ossification tended to form in regions where both the stimulus and *ρ*_*MSC*_ were moderately high. It is possible that the convex shape of the HO may be due to the level of cellular inflammation decreasing radially out from a certain point.

Only the Type 5 distal bulb like formation of HO was not able to be simulated here. This may be due to some limitations in the model in that systemic factors are not simulated. It is possible that in some cases HO is mechanically mediated but in others, genetic predisposition and other system factors such as hormonal changes primarily dictate the formation and distribution of HO. Another reason this formation was not seen may be due to the geometry of the model. In the distal bulb cases observed in the literature and available medical images, it appears that there is an extra medial distal flap of soft tissue.

Loading the model after the application of negative pressure dressings altered the final structure of HO suggesting that in some instances the morphology of HO is due to a combination of loading histories. This high sensitivity to loading, especially in the early stages of healing may shed insight into why such a vast range of HO morphologies is observed clinically. The simulation of tourniquet application resulted in no new bone elements forming in the soft tissue region after the first iteration. This may suggest that the short duration of pressure application from tourniquets does not instigate bone formation in soft tissue. However, Fig. 11 demonstrates a locally increased stiffness of the residual femur elements near the site of the tourniquet pressure application. This indicates that tourniquets may affect the periosteal lining. This finding is supported by literature stating that tourniquet application has been seen to increase periosteal bone formation in dog tibiae^62^. These changes within the tissues highlight the fact that other unaccounted for biological changes may occur under these local high stresses. These include factors such as tissue hypoxia from restricted oxygen which may influence cell differentiation^63^. Damage to endothelial and periosteal cells may also promote HO^9,17^.

There are a number of limitations in this current study, notably, experimental data are lacking to robustly verify these simulation results. The heterogenous nature of clinical HO may not have been captured fully in the classifications of morphology produced here. Also, a number of factors such as systemic cytokines and hormones, pH levels and available oxygen were not modelled. Only one loading case per simulation was applied. A future model could incorporate a combined weighted application of a number of loading cases as is used in other studies^64,65^. Precompression of the residual stump was not modelled and could potential influence the results as could three dimensional effects not modelled here. Other factors that could be incorporated in future studies include imposing an upper limit on stimulus for bone formation, and creating more granularity in material properties for different tissue types.

## Conclusions

The lack of effective prophylaxis for heterotopic bone, most of which involves adverse effects, means that an alternative, mechanically-based therapy is attractive. This study has shown that a mechanobiological simulation of heterotopic ossification implemented using adaptive finite element analysis can explain the formation of different characteristic shapes of heterotopic bone through changing mechanical parameters, suggesting that heteropic ossification is mechanically mediated. This study enables mechanical interventions to be conceived and trialled computationally.

## Data Availability

The full data presented in this paper is available on request from the corresponding author N. Rosenberg. Alternatively the data can be requested from the Imperial College London Spiral database with permission from N. Rosenberg.
